# Household visitation during the COVID-19 pandemic

**DOI:** 10.1038/s41598-021-02092-7

**Published:** 2021-11-25

**Authors:** Stuart Ross, George Breckenridge, Mengdie Zhuang, Ed Manley

**Affiliations:** 1 School of Geography, University of Leeds, Leeds, UK; 2 Centre for Advanced Spatial Analysis, University College London, London, UK; 3 Information School, University of Sheffield, Sheffield, UK; 4 The Alan Turing Institute for Data Science and Artificial Intelligence, London, UK

**Keywords:** Health policy, Applied mathematics

## Abstract

The COVID-19 pandemic has posed novel risks related to the indoor mixing of individuals from different households and challenged policymakers to adequately regulate this behaviour. While in many cases household visits are necessary for the purpose of social care, they have been linked to broadening community transmission of the virus. In this study we propose a novel, privacy-preserving framework for the measurement of household visitation at national and regional scales, making use of passively collected mobility data. We implement this approach in England from January 2020 to May 2021. The measures expose significant spatial and temporal variation in household visitation patterns, impacted by both national and regional lockdown policies, and the rollout of the vaccination programme. The findings point to complex social processes unfolding differently over space and time, likely informed by variations in policy adherence, vaccine relaxation, and regional interventions.

## Introduction

Human behaviour has been well established as a critical factor in the effective mitigation of virus transmission^1^ with the mixing and mobility of individuals understood clinically as paramount to causing COVID-19 to spread^2–4^. Since early 2020 local and national governments have imposed Non-Pharmaceutical Interventions (‘NPIs’) to control the virus, including ‘stay-at-home’ orders and social distancing requirements. England has suffered successive NPI restrictions over a sustained period, punctuated by multiple ‘national lockdown’ periods spanning March–May 2020, November–December 2020, and January-March 2021, where health restrictions were the same nationally (see Supplementary Materials 1). These and intervening periods offer the opportunity to study the varied effects of human behaviours including ‘lockdown fatigue’^5^ across a large population sample, through the proxies of measured human mobility and its subsets over time.

Mobile phone data provide a valuable source of information for monitoring the aggregate dynamics of human mobility behaviour. Insights from mobile phone data have been used historically for urban planning and management^6,7^, with research continuing to call for its use in the monitoring of changing public space use during the COVID-19 pandemic^8–10^. However, public health professionals are now also calling for the use of this ‘opportunistic’ mobile phone mobility data under COVID-19, arguing it could play a role in analysing the effectiveness of ‘lockdown’-style NPIs in their ability to reduce human activity^4,11–14^.

A body of research has responded to this call^15^, examining a multitude of mobile phone mobility data to indicate the extent to which populations are responding to the imposition and lifting of COVID-19 ‘lockdown’ measures^16,17^. The dominant form of activity measurement has been temporally and spatially aggregated forms of overall mobility (e.g., Google Mobility Trends Reports), with these studies claiming these measures act as a suitable proxy for COVID-19 transmission opportunities. A study on UK policy ‘adherence’ during the first national lockdown used a combination of mobile phone data from O2 and GPS smartphone data from Facebook to examine mobility changes through the number and distance of ‘journeys’ identified^12^. It concluded that compliance to the stay-at-home order in the UK was ‘high’ and ‘geographically-even’ as of late May 2020. However useful, these studies examining metrics for overall mobility extent may fail to capture behaviours driving the actual mechanisms of COVID-19 virus transmission^16^, which is now known to vary significantly between, for instance, indoor and outdoor environments^18^.

This study proposes a novel framework for the identification of visits by de-identified individuals to non-home households. This metric is derived through mobile phone trajectory data, and by extracting mobility behaviours of this type, we are able to derive an indicator of ‘household mixing’ by location and time. While the transmission of COVID-19 is a highly complex social phenomenon, we propose that this measure can yield an additional explanatory factor in understanding transmission, and subsequently inform policy design. The approach we propose uses passively collected and GDPR-compliant GPS trajectory data, collected through smartphone apps from opted-in anonymous users who provided informed consent to data collection for research purposes, by the data company, Cuebiq. Our study focuses on England prior to and during the COVID-19 pandemic in 2020 and 2021. Cuebiq mobility data have been used elsewhere to analyse mobility under COVID-19 amongst US metropolitan areas and states^19,20^, and in the UK, Italy, Colombia, Mexico and Indonesia^21–25^. As part of this study, we query Cuebiq data through a privacy-preserving platform to generate aggregate insights into household visitation patterns, while also further addressing the validity of these data for estimating population level indicators.

Our period of analysis means we address issues of adherence to policy, both during harsher periods of ‘lockdown’ (which varied in their severity in relation to household visits, see Supplementary Information 1) and the effect of the vaccine rollout on behaviour. On these points, to date, the issues of household mixing and policy adherence have been addressed only indirectly. Fierce debates continue over the causes and extent of population-wide non-adherence in the UK^5^. Although some survey data indicate that over 90% of the British people have consistently adhered to social distancing most of the time^26^, other survey data indicate that non-adherence to ‘stay-at-home’ orders is far higher. Hills and Eraso^27^ find that ‘contrary to a perceived sense of people’s adherence’ 92.8% of London residents surveyed did not adhere to all social distancing rules, with 48.6% engaged in ‘intentional non-adherence’. Other studies have sought to identify the causes of non-adherence^28–35^ although their collective findings are highly inconsistent and often contradictory, possibly given that causal factors may change over time^34^. We join a multitude of public health researchers in proposing that through the use of passively collected anonymous mobility data, we can overcome the degree of social desirability bias that typically mediates the self-reporting methods used historically to indicate policy adherence^27,28,30,36–39^, meeting the call that “future research should focus on assessing adherence with objective measures to minimize the likelihood of biased reporting”^39^. In doing so using house visits and particular case studies, we hope that this can further address the “[current] lack of empirical evidence to support the fact that there is significant ‘fatigue’ around adherence”^38^ to COVID-19 restrictions in the UK, helping to accurately inform health policymakers devising the English response.

## Results

We consider the extent of household visitation in two main ways—its evolution over the course of the COVID-19 pandemic across the whole of England, and in response to policy and medical interventions; and its variation over space, reflecting regional and land-use trends and regional policy contexts. The definition of terms and methods for calculating household visitation rates is detailed in the *Materials and Methods* section.

### Evolution of ${H}_{England, t}$

**Figure 1:**
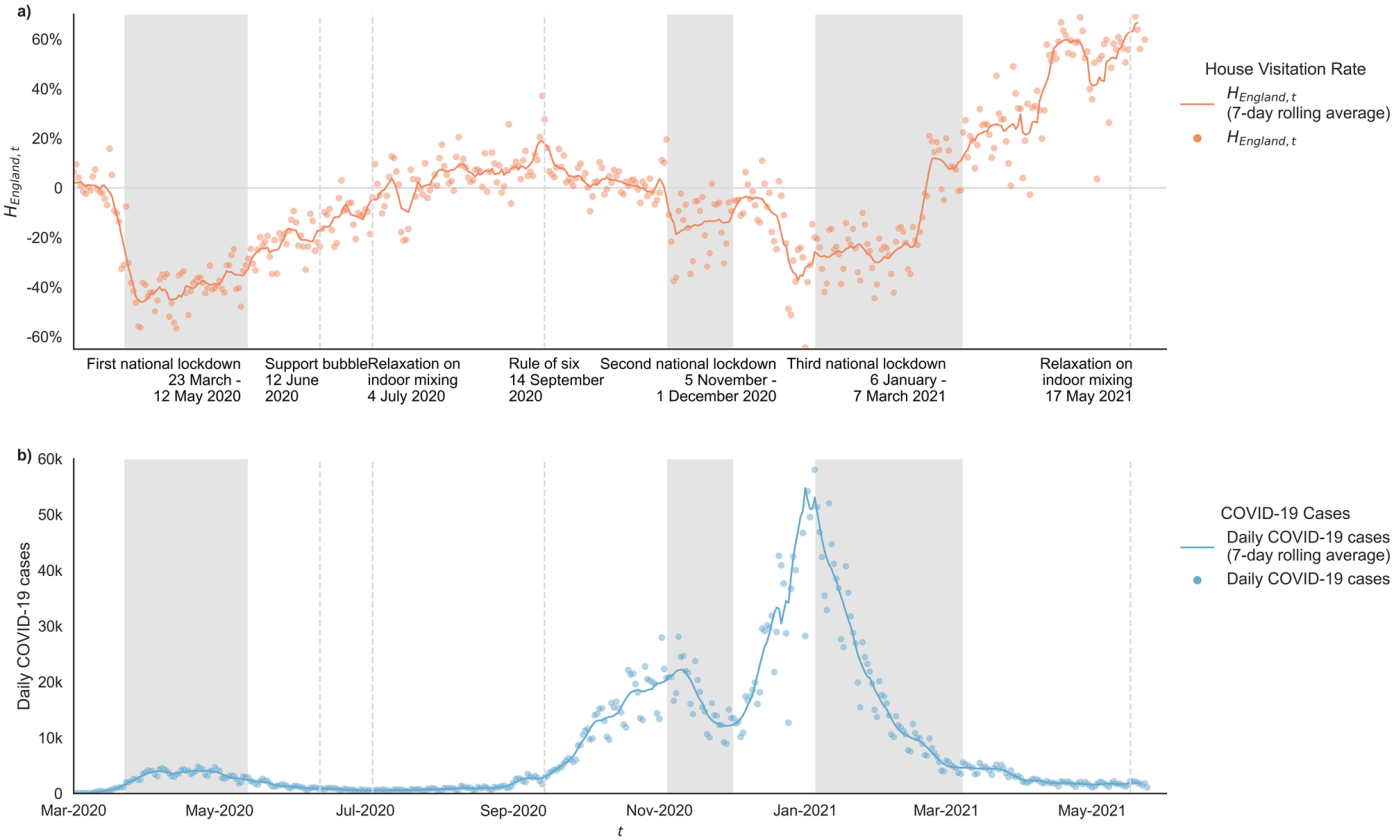
Changes of (**a**) ${H}_{England, t}$ and (**b**) daily new COVID-19 cases in England between March 1st 2020 to May 24th 2021. For each day, the national ${H}_{England, t}$ is aggregated from the ${H}_{l, t}$ of each local authority. Accounting for the day of week effect, ${H}_{England, t}$ is also illustrated with the rolling 7-day average. New COVID-19 cases are the number of cases by specimen date. Time periods or time points where indoor mixing rules were changed were marked according.

We begin by illustrating the variation in ${H}_{England, t}$ at the national scale between March 2020 and May 2021 (Fig. 1a). The largest reduction in ${H}_{England, t}$ occurs shortly after the first national lockdown came into effect on 23rd March 2021 (largest daily decrease on 24th March; − 22.8%, lowest level of ${H}_{England, t}$ reached was − 56.4% on 29th March). This reduction aligns with other mobility metrics (see Supplementary Information 2 highlighting the significance of the initial lockdown in restricting all forms of mobility behaviour. Yet, despite the continued imposition of the national lockdown policy during this period, as the first lockdown ends, there was a steady increase of ${H}_{England, t}$*.* The first easing of lockdown policy has an immediate significant impact on house visitation (${\Delta H}_{l, t}$ between 0.3 and 26.9%, $p$-value = 0.0439). This increasing trend continues through the Spring of 2020, seemingly unaffected by introduction of ‘support bubbles’ (legally enabling mixing between paired households in certain circumstances) on 12th June ($p$-value = 0.6846). Household visitations continue to increase, reaching baseline levels (i.e., ${H}_{England, t}$ = 0%) 8 weeks after the first lockdown ended on 5th July and taking another 8 weeks to a 2020 peak (${H}_{England, t}$ = 37.1% on 13th Sep). These increases align closely with the relaxation of the rules on indoor mixing on 4th July 2020 (${\Delta H}_{l, t}$ between 0.6 and 27.3%, $p$-value = 0.0407), although appear to decrease with the operation of the ‘Eat Out to Help Out’ scheme during August, and had a dual purpose of supporting the hospitality industry and promoting social interactions in ‘safer’ public settings (${\Delta H}_{l, t}$ between − 25.6% and − 6%, $p$-value = 0.0015). The ${H}_{England, t}$ declines through September as limits on gatherings (‘Rule of Six’) and regional health regulations were introduced, in response to increasing case numbers (see Fig. 1b).

A second national lockdown was imposed on 5th November 2020. Despite a slowly declining trend during October, we observe sharp increases in ${H}_{England, t}$ in the days prior to the lockdown commencing (yet after its announcement on 31st October 2020). For LTLAs not in tier 2 or 3, ${\Delta H}_{l, t}$ is not significant ($p$-value = 0.2268). This pre-lockdown peak resembles other anticipatory highs in mobility activity observed elsewhere in the immediate period before new COVID-19 restrictions are introduced^16,21^. House visits remain consistent throughout the second lockdown (${\overline{H} }_{England,NL2}$= − 15.28%), yet above those levels observed during the first lockdown (${\overline{H} }_{England,NL1}$ = − 39.33%), with a quicker return to baseline activity on 2nd December, contrasting observations from the first lockdown.

In December a rapid rise in cases is observed in part due to the rapid transmission of the B.1.1.7 variant (the so-called ‘Kent variant’, or variant Alpha). In response, new stay-at-home regional restrictions on activity were announced on 19th December 2020, leading to stronger reductions in house visitation (${H}_{England, t}$ = − 21.5%), although still remaining above the levels of the first lockdown. These trends are reinforced by the imposition of a third national lockdown on 6th January 2021, causing ${H}_{England, t}$ to remain consistently low until mid-February 2021 (${\overline{H} }_{England, t}$ = − 26.22%, where $t$ runs over the dates between 6th January and 14th February). At this point a large spike increase in activity is observed 2021 (${\overline{H} }_{England, t}$ = 2.1%, where $t$ runs over the dates between 15th January and 7th March), coinciding with announcements that the most vulnerable citizens had been vaccinated on the 14th February 2021. We observe gradual increases in ${H}_{England, t}$ despite household mixing remaining heavily restricted until 17th May 2021.

### Regional variation

**Figure 2:**
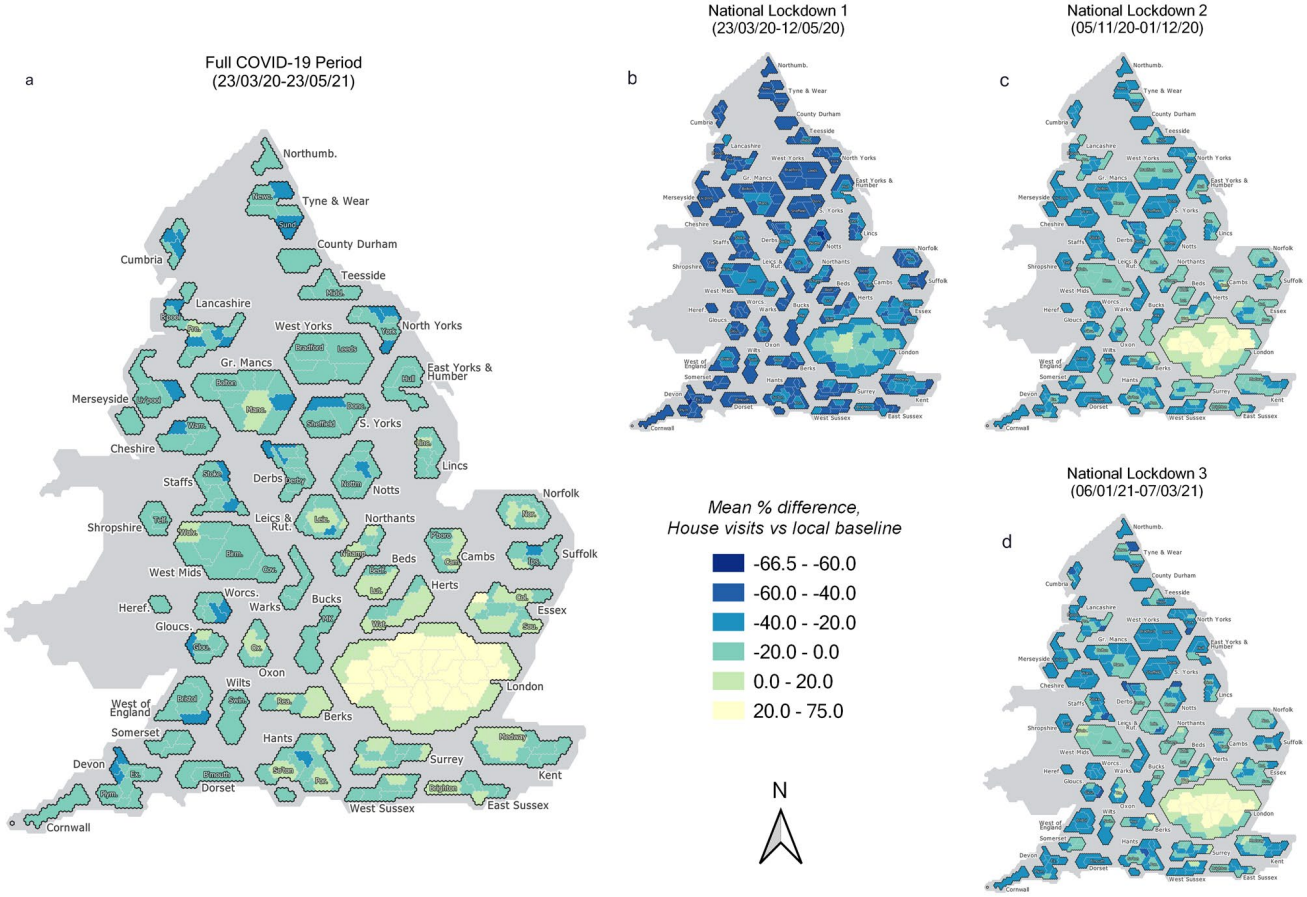
Hexagon-based cartogram distribution in ${H}_{l, t}$ during (**a**) the England COVID-19 period, and (**b–d**) three national lockdowns. Local authority boundaries are resized by population count to enable greater visibility in more highly dense regions (i.e., cities), and were prepared by the UK House of Commons Library.

The progression of the virus exhibits strong spatial dependence, and as such restrictive policies were imposed variously at regional scales in England to counteract these regional trends. To explore insights into local patterns of household mixing and the effect of regional policy, we illustrate the spatial distribution of ${H}_{l, t}$ across the entire study period and three national lockdowns (Fig. 2) which shows some clear geographic differences in the extent of household mixing. These cartogram maps—which show local authority regions with size adjusted for population count and arranged according to approximate geographic location—highlight consistently higher levels of ${H}_{l, t}$ in the London and South-East regions. Outside of these regions, higher measures of ${H}_{l, t}$ are observed in some urban areas—including Manchester (${H}_{Manc, t}$ = 2.1%), Cambridge (${H}_{Camb, t}$ = 10.9%), and Leicester (${H}_{Leic, t}$ = 3.6%). The lowest levels of ${H}_{l, t}$ are found in rural authority areas, such as North-East Derbyshire (${H}_{NEDerbys, t}$ = − 27.9%), West Devon (${H}_{WDevon, t}$ = − 25.2%), and Mid Suffolk (${H}_{MidSuf, t}$ = − 24.5%). The variance in ${H}_{England, t}$ observed in Fig. 1a during the different national lockdown periods is demonstrable at the regional scale in Fig. 2b–d. While the regional variation in ${H}_{l, t}$ remain broadly similar, evidence of a reduced strength of reduction is repeated.

**Figure 3:**
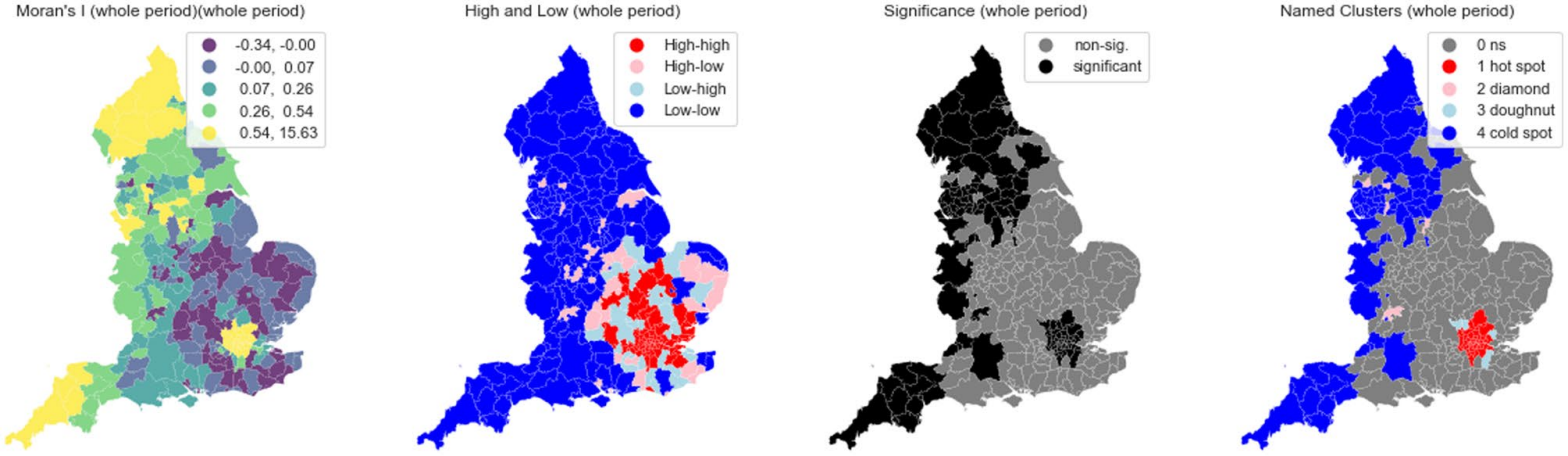
Spatial measures of Local Moran’s I and Local Indicators of Spatial Association^40^, based on eight nearest neighbours for the whole study period.

We find statistical evidence for regional variation in these activity through calculation of spatial clustering. Figure 3 describes location of clusters of higher and lower rates of ${H}_{l, t}$ as well as identifying regional outliers. These figures more accurately highlight areas of London and the South-East as significant (95% threshold) hotspots of high levels of ${H}_{l, t}$ and areas of the South West and North West as areas of significant coldspots. The figures highlight outliers in regional trends too—for example, St Albans and Kingston-upon-Thames show lower levels of ${H}_{l, t}$ relative to the wider trends in the South-East. When reviewed, these trends were consistent across each national lockdown.

**Figure 4:**
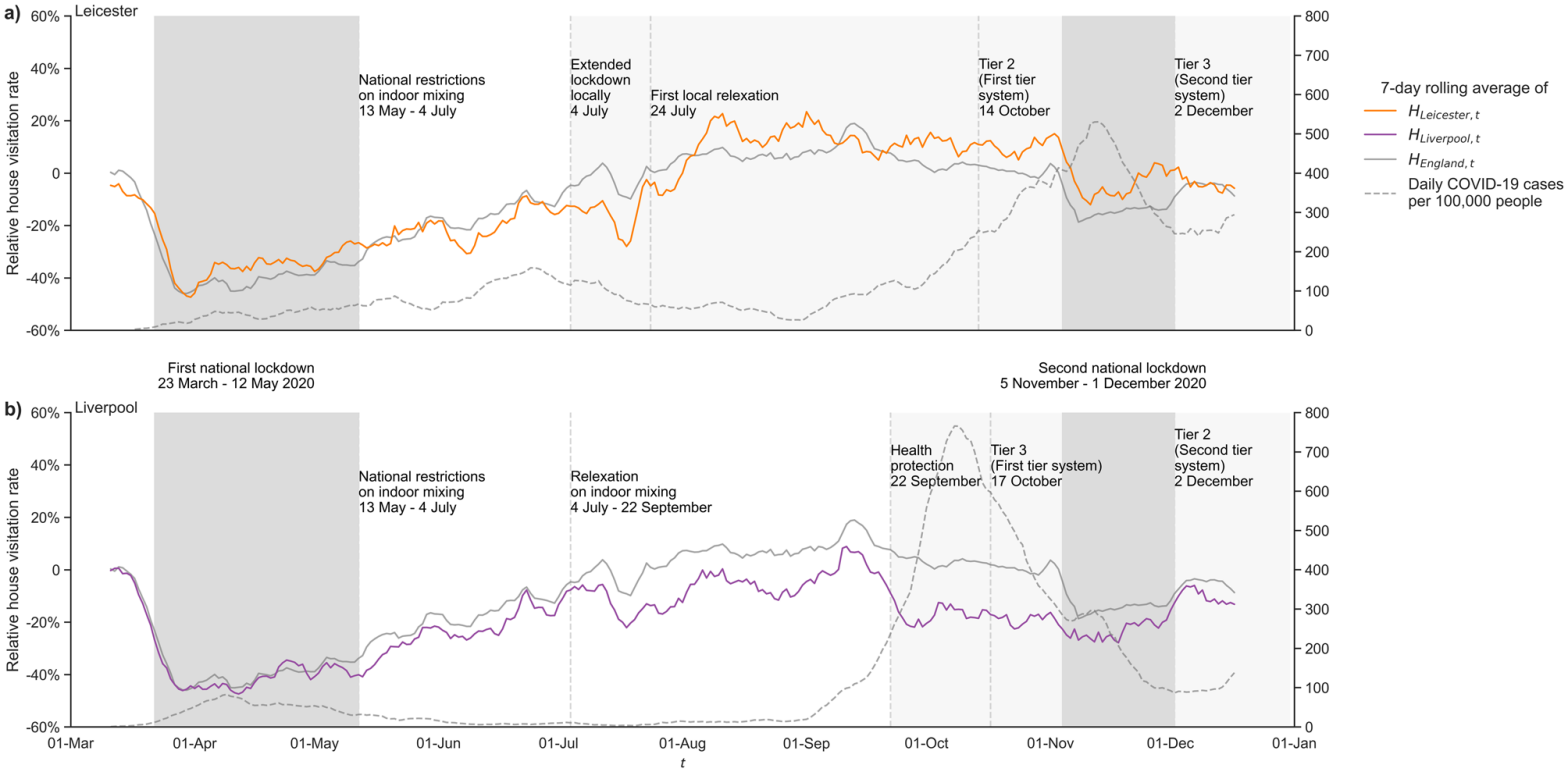
Change in ${H}_{l, t}$ in (**a**) Leicester and (**b**) Liverpool from March 2020 to December 2020, where regional health protections regulations were implemented.

While the geographic scale reveals spatial trends in ${H}_{l, t}$, we can gain more insight into regional variation through exploring regional trends over time. Figure 4a,b show variation in ${H}_{l, t}$ for two local authorities—Leicester and Liverpool—that had heavy local restrictions imposed outside of periods of national lockdown (see Supplementary Material 1).

In Leicester, higher than average case numbers led to imposition of a regional lockdown policy on 4th July 2020. Up to this point, ${H}_{l, t}$ had been tracking the national ${H}_{England, t}$ in gradual increases following the easing of restrictions after the first lockdown, yet the regional lockdown marks a point of divergence. Nevertheless, despite similar policies being imposed at this point, levels of ${H}_{l, t}$ barely reach the low points of the first lockdown (${\overline{H} }_{Leicester, NL1}$ = − 34.68%), suggesting reduced efficacy at least in terms of household visits. At the first easing of these regional restrictions on 1st August 2020 (reopening of places of worship, pubs, restaurants, cafes, and hairdressers) levels of ${H}_{l, t}$ rose sharply to above the national average and regional baseline levels, despite household mixing still being banned.

Liverpool tells a slightly different story. When regional restrictions were imposed on the 22nd September 2020, measures of ${H}_{l, t}$ were relatively low and reducing, and plateaued until the end of the second national lockdown. This could suggest a stronger adherence to regional policy but may have been reinforced by increasing residual case numbers (which showed a more significant rise than in Leicester).

### Vaccinations

**Figure 5:**
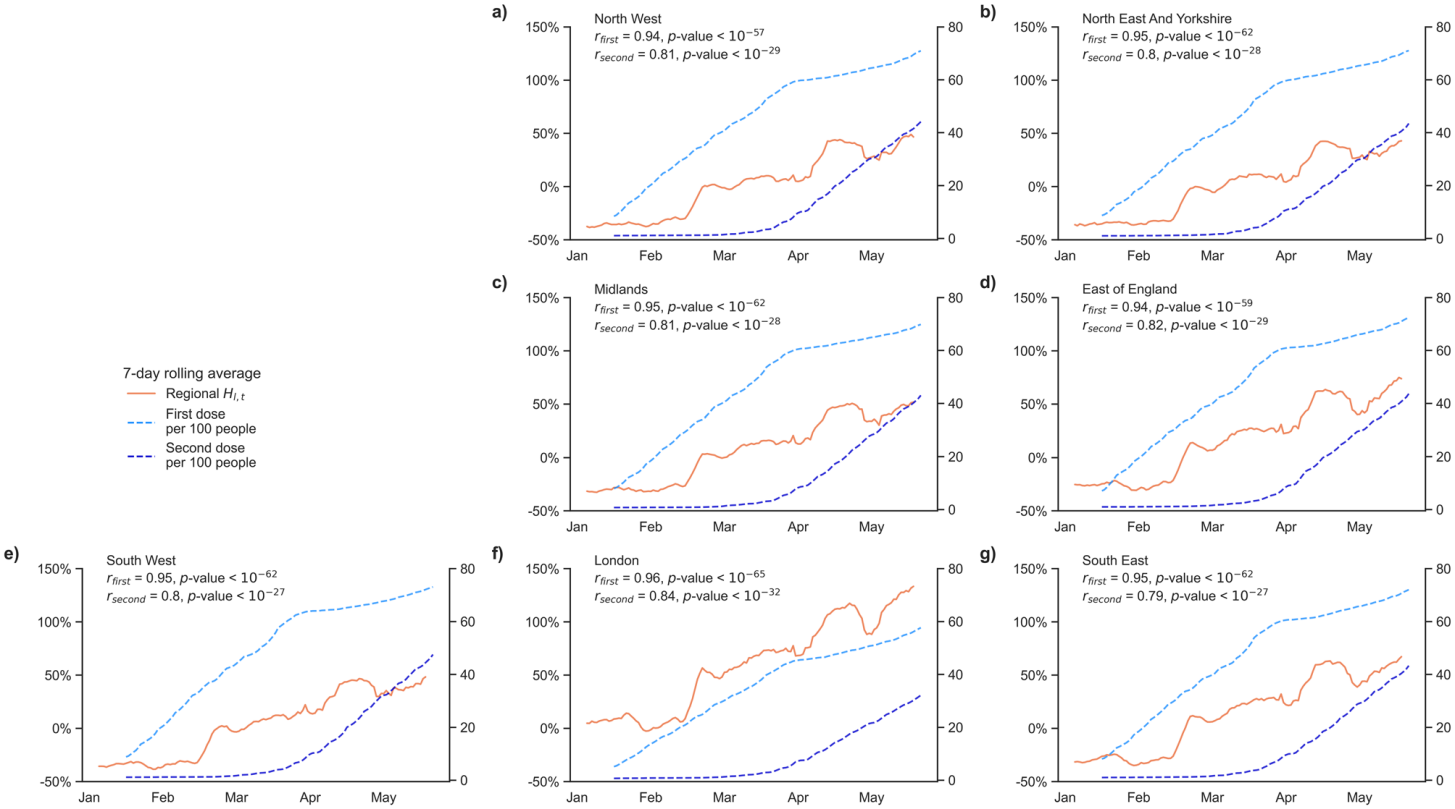
Change in ${H}_{l, t}$ in England regions from January 2021 with number per 100 people who received first or second dose of COVID-19 vaccine. (**a–g**)**,** data on vaccinations has been released at the scale of UK regions since mid-January. Significant positive correlations are observed for all regions between regional ${H}_{l, t}$ and number of first dose or second dose of vaccinations.

The increase in household visits from mid-February 2021 appears to align with the rollout of vaccinations across England. Following an initial phase of vaccination of vulnerable populations, the programme proceeded by age cohort. England has offered vaccine to the top four priority groups by 15 February and started inviting people aged 65–69 (${\Delta H}_{l,t}$ between 1.4 and 23.3%, $p$-value = 0.027). Figure 5 shows how the vaccine programme moved from a primary focus on first dose vaccinations, to provision of second doses from April onwards. The figure also reinforces the notion of a broad correlation between vaccinations and the regional house visitation rate, that is consistent across all regions of the UK. The increases in household visits are shown at a time where indoor household mixing was heavily restricted, and only relaxed on 17th May 2021.

## Discussion

The principle that human interaction in confined spaces is fundamental to the propagation of COVID-19 has been well established, as has the notion that aggregate mobility data can point to instances of risky behaviour associated with its transmission. In this paper we have addressed the potential for these data to uncover population-scale patterns of adherence in household visitation policy. This is a type of mobility behaviour that requires longitudinal analysis of anonymised individuals, using data that are not widely used or available to public health professionals and academics; it therefore raises important issues for future public health policy and ethics.

The trends uncovered through these analyses are significant for a number of reasons. There is demonstrable temporal and spatial variation in household visitation during the course of the pandemic, but the nature of causation is less clear. Broadly we observe a strong reduction in household visitation during the first national lockdown in England, but over the course of mid-2020, despite strong public caution, residual cases, and policies in place to limit household visits, we observe a steady increase in household visit events. Pre-COVID baseline levels of household visits return in July 2020. The later imposition of household mixing bans at regional level again indicates some impact on these trends, but the extent of household visitation never again reaches the lowest levels observed in March 2020. This supports speculations^41^ that non-adherence is driven by the novelty of the threat, and that multi-lockdown fatigue is a concrete phenomenon.

More concerning in terms of our observations are the significant increases in household mixing, particularly during the first half of 2021, that likely contravene policy restrictions. The trend of increased visitation behaviour is first identified following relaxations of policy at regional (e.g., Leicester) and national scales (e.g., following the second national lockdown). The indication of these findings is that people are effectively ‘overcompensating’ for their confinement through increased social activity. In 2021, we see significant increases in household visitation that aligns with the timing of the vaccine rollout in England. During this period these drivers may be reinforced by a new perception of safety through vaccination (and of vaccination of others), despite evidence and guidance to the contrary.

These patterns of household visitation show important spatial variation. In general, the London and South East regions demonstrate higher rates of household visitation during national lockdown periods. As we will note below in summarising the methodological limitations, due to the density of populations, the actual rates of household mixing may be higher than what we report here. The trends point to consistently higher rates of policy non-adherence, perhaps wrought by a perceived necessity of interaction (by virtue of lifestyles), or a failure of policymakers to speak to particular populations.

An important message from these analyses is that adherence to restrictions on household visitation has often been inconsistent over space or over time. While policy appears to have some effect on household visitation patterns, the effect has weakened over time, and has always been less well adhered to in some regions. The notion that social policy can be simply ‘turned back on’ is severely diminished by these findings. The data also point to a public reversion to ‘normal’ household visitation patterns occurring at different rates—cautious (e.g., following first lockdown) to quick (e.g., following the second lockdown)—and prompted by different sentiment—a relief in response to lockdowns ending, or the perception of safety prompted by the vaccine rollout. These trends point to the complex and multifaceted nature of ‘behavioural fatigue’^25^.

The study demonstrates the benefits of engaging mobility data in different ways to uncover larger scale patterns of human behaviour. These data have played an important role in the management of the pandemic, but their application with greater sophistication could enable even wider utility. Nevertheless, there are a range of important technical and ethical caveats that must be considered in relation to the wider utilisation.

A principal concern of this type of analysis relates to the representativeness of the dataset to reflect wider population trends^4,42^. Yet recent evidence suggests smartphone data may be more representative than previously recognised. A leading study on UK lockdown ‘compliance’ showed high similarity between UK mobility levels in early- to mid-2020 when comparing Facebook GPS app data with CDRs from network operator O2^12^. As we describe in Supplementary Information 3, we did not uncover *prima facie* grounds for these concerns in our particular context and dataset.

However, given that our data are anonymised to ensure privacy, we are blinded from a comprehensive understanding of the demographic, socioeconomic, and contextual factors governing the observations of our cohort^43,44^, which may be skewed relative to the wider population. Biases are potentially introduced through the requirement of our sample participants to own a smartphone, and within this group to also use Cuebiq partner apps with voluntary location-sharing permissions granted^13,20^. Dependent on hardware and software settings, we are then also potentially filtering down to people active on the Cuebiq partner app and/or those moving around physically^4^, which may also cause an inconsistent and/or declining user base over time^22^. In our study, we observe a ‘rate of attrition’ in our sample that is suggested of this (see Supplementary Information 4), as people become inactive on the apps, as they change device, update their phone operating system^45^, or change location-sharing permissions. Our study is also limited in using smartphone mobility data as a proxy for individual human mobility. Ultimately, we are analysing movement of the device, not people.

The ‘new mobilities’ era, characterised by ‘digital traces’ left behind from individual mobility^46^, has finally realised its long-promised social value in the monitoring of epidemics and their management under COVID-19. Nevertheless, ethical and privacy concerns punctuate these COVID-19 use cases^47–49^. As an unprecedented global health emergency in modern society, COVID-19 has asked new questions about the ‘responsible-use’ cases of individual-scale movement data. Although the uptake of ‘tracking’ apps has been poor amongst some national geographies^9^, phone apps collecting personal location data to aid the epidemiological response to COVID-19 are generally accepted by the public^49^. The collection of location data by Cuebiq is premised upon consent that is freely given, specific, informed and unambiguous. All data are anonymised before it is accessed by researchers, with strict aggregation requirements in time and space imposed to qualify datasets for export, to protect privacy and ensure the conditions of consent remain fulfilled.

Regarding the treatment of these data in this study, there are important limitations that raise further caveats around the interpretation of results. One specific issue relates to the removal of workplaces and points-of-interest prior to the identification of household visits, as documented in the Methods section. By introducing strict limits on visits proximal to these locations, we preclude household visits around mixed-use developments (e.g., where a shop is next to a visit location) and public transportation infrastructure (e.g., bus stops). This is an important step in data filtering, but has implications for certain regions, and one can expect this results in underestimation of household visits in denser, urban areas. Likewise, visits to neighbours within multi-story buildings will be missed by virtue of these points being interpreted as activity at ‘home’. Further analysis would be beneficial in relation to the identification and verification of workplace visitation patterns, and some collection of representative labelled work shift data would be useful in this regard. There are equally other areas where our interpretation of ‘high’ levels of household visitation requires tempering—specifically in cases where visits may have been to gardens, which lie within the threshold for a household visit but would not be deemed a policy breach after June 2020. Further refinement of the methods would require access to accurate data on household greenspaces.

The patterns presented in this proof-of-concept study, as well as the limitations we have raised, point to areas for further exploration. The specific focus on household visitation, and the nature of its variation over time and region, suggest it is a factor worth exploring within future models of policy response. Further mixed methods approaches are required to help attribute causation relating to higher rates of household visitation, but the regional and temporal variation in propensity to make household visits could provide a useful additional predictor of virus evolution. If a broader base of evidence linking household visitation to virus transmission is established, the *h-index* proposed here provides a useful potential indicator in the specification, communication, and monitoring of public health policy. The regional and temporal comparative analysis we have proposed here suggests that policy has had a differential effect, yet it is challenging to unpick exactly how and why. Policymakers, officials, and citizens can likely provide the greatest insights here, but they require the tools on which to make those evaluations.

## Materials and methods

### Data sources

#### GPS data

This research used a dataset containing 1.58 billion GPS records from more than 1 million anonymised and opted-in mobility phone users in England over 17 months, from 6th January 2020 to 24th May 2021. The raw data contain an anonymised ID of a user, a GPS-derived location containing a latitude and longitude, an accuracy measures for the location in metres, and a timestamp of when the user was at the location. The data was provided by Cuebiq, who collect data from opted-in, de-identified users of smartphone apps who have provided informed consent for their anonymised data to be used for research purposes. Data collection and processing is fully GDPR compliant. The data was accessed by researchers via the Cuebiq Workbench platform, an auditable sandbox environment that allows access for querying of data and generation of aggregate, privacy-preserving outputs. The sandbox enables the creation of aggregate data at regional levels, without the ability or need to create individual-level outputs. In this study, only GPS records with less than 100 m accuracy and on land are considered, leading to a final dataset of 1.06 billion records from 984,000 users.

The geographic scope was limited to England for two primary reasons. The first is to constrain our study to one set of national public health policies. The public health response in England is led by the UK Government and Public Health England, with other UK nations managing their own policy programmes. The policies adopted in England are similar to many other countries (e.g., regional and national lockdowns, school closures). The second was that while the data cover the entire UK, there are clear reductions in sample size when looking beyond England.

#### *Non-pharmaceutical intervention *(*NPI*)* data*

We sourced England’s national and regional NPIs from the UK House of Commons library.

#### COVID-19 case and vaccination data

We use COVID-19 new cases data by specimen date and weekly number of vaccination numbers from the ‘Coronavirus in the UK’ dashboard and the weekly COVID-19 vaccinations report. To control for daily fluctuations of COVID-19 new cases data, we smooth the time series using the seven-days rolling average approach.

### Estimation of visitations

The visitation points of our cohort form the foundation of the analysis. The calculation of visitation points was made through a three-stage clustering methodology.

In this first stage *daily candidate visitation clusters* were established through spatial clustering for each user. These clusters made use of ‘stop point’ data, which capture device locations moving at 2 miles per hour or less. The clustering of these points was carried out using Density Based Spatial Clustering of Applications with Noise (DBSCAN), which has been well established in its use for determining a visitation point points from trajectory data and has the benefit of isolating visitation points from other points, classified as noise. The spatial clustering parameters used in this study were $\epsilon$ = 33 m and $mi{n}_{p}ts$ = 3. The implementation of DBSCAN in the Python package scikit-learn was used. A centroid for each cluster was calculated.

In the second stage *daily candidate visitation clusters* were further assigned with *number of visits* by findings temporal gaps within a spatial cluster. For each user, a daily stopping threshold is calculated as the smaller of the following two values: 30 min and the standard deviation (SD) of the time increments of the user’s points respectively (accounting for variable interpersonal sample rates due to movement patterns, device characteristics, etc.). *Number of visits* were calculated as one plus the number of temporal windows of greater than daily stopping threshold between subsequent points in a daily candidate visitation cluster.

The third step in determining daily visitations was to filter out very short visits, by setting a minimum stay time for each daily visit. For this stage we used a 15-min minimum period. This 15-min window helps to remove any remaining noise from insignificant stops such as traffic jams or picking up a package.

### Estimation of ‘home and work’ locations

‘Home and Work’ locations were established as the two most frequently visited locations by each individual within a 2-week baseline period, calculated on a rolling basis at the beginning of each month. By recalculating these locations each month, we were able to maintain a large sample size (by adding new users) while allowing for home moves and changes in circumstances (e.g., working from home).

‘Home and Work’ locations were calculated using DBCSAN ($\epsilon$ = 33 m, $mi{n}_{p}ts$ = 3) on a similar basis as visitation points, except using the centroids of visitation clusters over a full 2-week period. The two clusters with the highest sum of number of visits assigned were set as home and work in order.

All *daily visitation clusters* intersecting within 30 m of a ‘Home and Work’ cluster centroid were removed from the visitation list for that user.

With ‘Home and Work’ locations in place for a set of users, a further reduction of our user subset was undertaken to remove users observed on fewer than four different days and those with fewer than 10 destinations. Users with extremely high stopping frequency were classified as ‘mobile workers’ and also excluded from this analysis.

### Removing POIs and greenspace

Visitation clusters were removed if they fell within a 30-m buffer of an established Point of Interest (POI). POIs were taken from the Ordnance Survey POI layer, that provides a comprehensive collection of potential visit locations throughout the UK ranging from churches to restaurants to sports stadiums. A limitation of this step is that potential household visitations are more likely to be removed in areas of mixed land use. Households that are close to or share the same building footprint (e.g., within the same tower at different levels) as a POI would be ignored in this study. It can be expected that this has a stronger effect in denser urban areas.

Ordnance Survey also provided greenspace polygon data, outlining all parks and woodland in the UK. Destinations were also removed if they fell within one of these greenspaces.

### Validation of household visitations

Following the removal of individual ‘home and work’ locations, and POI and greenspace visits, a remaining set of visits points were deemed *unclassified visits* locations. To provide a final validation of the household visit estimation, *unclassified visits* from the entirety of 2020 were extracted, and compared against the Ordnance Survey AddressBase dataset, which records the location of all residential addresses in the UK. This established that 89% of unclassified visits were within 50-m of a residential building. As a result, the remaining 11% of visits (i.e., those above 50-m from a residential building) were removed from the remaining analysis. The final dataset of visits is classified as *household visitations*.

It should be noted that our definition of household visitation does not preclude visits to private gardens, which was permitted under the policy guidance at various points during the pandemic.

### Active users

To explore the spatial variation of household visitations, we further grouped the users with ‘Home and Work’ locations into each LTLA ${l}$ by their home location. England’s LTLA geometry data is obtained from the open geography portal. We then calculate the number of active users for each day ${t}$ and each LTLA ${l}$, ${N}_{l, t}^{au}$. Active users are users belonging to LTLA ${l}$ and have at least one *daily visitation* on day ${t}$. A further subset of active users who have *house visitations* are extracted, and the size of this subset is referred as ${N}_{l, t}^{hv}$. The LTLA Isle of Scilly is excluded as it has less than 10 active users, failing the minimum statistical disclosure control. In total, 313 LTLAs in England were included in the further analysis.

### Household visitation rate ${H}_{l, t}$

The central idea of creating this rate is to compare the varying household visitation rates by region with a baseline level set prior to the COVID-19 pandemic. Two steps are applied here. We first determine the *household visitation rate*,${V}_{l, t}$, for each day ${t}$ and each LTLA ${l}$, where ${V}_{l, t}={N}_{l, t}^{hv}/{N}_{l, t}^{au}$ . Then, we normalise this rate by the pre-pandemic baseline, giving ${H}_{l, t}={(V}_{l, t} - {V}_{l, d}^{Baseline})/{V}_{l, d}^{Baseline}$. The baseline, ${V}_{l, d}^{Baseline}$, is calculated through averaging the daily *household visitation rate* across the 8 weeks before the pandemic (from 13 January to 2nd March 2020) by day of week ${d}$ for each LTLA ${l}$, accounting for the periodicity of social activities (e.g., greater visit count between Friday and Sunday) and spatial heterogeneity between LTLAs. For specific LTLAs, we name it directly with the LTLA name, e.g.,${H}_{Leicester, t}$.

To compare the spatial variation of ${H}_{l, t}$ , we aggregate ${H}_{l, t}$ at regional and national levels. For example, we refer to ${H}_{England, t}$ as England’s house visitation rate on day ${t}$, yielding ${V}_{England, t}=\sum_{l}{N}_{l, t}^{hv}/\sum_{l}{N}_{l, t}^{au}$ , where $l$ runs over the 313 LTLAs in England, and calculate ${V}_{England, d}^{Baseline}$ by averaging ${V}_{england, t}$ in the eight pre-pandemic weeks by day of week ${d}$.

To compare the temporal changes of ${H}_{l, t}$, we further take the mean of ${H}_{l, t}$ over a fixed period at an aggregated level. For example, we refer ${\overline{H} }_{England,NL1}$ as the mean of ${H}_{England, t}$ during the first national lockdown where $t$ runs over the duration of the first national lockdown in England.

### Estimating impact of events

To test the immediate impact of events (e.g., new NPIs or policy announcements) on ${H}_{l, t}$ we calculated $\Delta {H}_{l, t}$, the differences between ${H}_{l, t}$ 1 week before and 1 week after the event took place in the LTLAs through a regression discontinuity analysis. By using a 15-day window, we are able to smooth the day of week effect on mobility while limiting the impact from events which occurred outside this window. A linear fixed-effect model is defined as,

$${H}_{l, t}= {\beta }_{0}+{\beta }_{1}{D}_{t}+{\beta }_{2}{E}_{t} +{\beta }_{3}{D}_{t}{E}_{t}+{\alpha }_{l}+{\epsilon }_{l,t}$$

where the variables on the right-hand side of the equality are as follows: $D$ is the indicator variable of the post-event period, namely, ${E}_{t}$ takes value 0 before the event and 1 afterwards. ${D}_{t}$ is the index of the day in a 15 day time window centred on the event, and runs from − 7 to 7, ${D}_{t}{E}_{t}$ is the product of the previous two features. We fit this model with ordinary least squares (${\epsilon }_{l,t}$ are independent samples from a Gaussian with fixed variance). ${\alpha }_{l}$ is the individual entity effect of each LTLA, which is a priori unknown. We call ${\beta }_{2}$ the Local Average Treatment Effect $\Delta {H}_{l, t}$, and statistically test for this value being different from 0 using a standard t-test for the parameters of linear regression. Clustered standard errors by day and LTLA are applied. The Python package *linearmodels* was used for implementation.

### Significance statement

Human behaviour and interaction in enclosed spaces has been fundamental to the transmission and spread of COVID-19, and many public health policy responses focused directly on reducing these interactions. Yet our understanding of the extent to which these interactions have continued to take place is limited. This research proposes a new indicator of COVID-19 policy adherence. Using a large mobility dataset, collected over the course of the pandemic, we measure how household visitation has evolved. We are able to assess the relative levels of variation in policy adherence over space and time. The measure provides a new tool in how we develop, deploy, and monitor COVID-19 policy effectiveness at regional and national scales.

### Ethics declaration

This research has been approved by the University of Leeds Ethics Board.

## Supplementary Information

Supplementary Information.

## Data Availability

The data that support the findings of this study are available from Cuebiq through their Data for Good programme, but restrictions apply to the availability of these data, which were used under licence and so are not publicly available. All methods were carried out in accordance with relevant guidelines and regulations.

## Code availability

Code used to produce these analyses is available on GitHub at https://github.com/mdZ01/Covid-Household-visitation.
