# Supplementary material for: Household visitation during the COVID-19 pandemic
Source: Sci Rep. 2021 Nov 25;11:22871. doi: 10.1038/s41598-021-02092-7 (PMC8617191; doi:10.1038/s41598-021-02092-7)

**Supplementary Information for**

**Household visitation during the COVID-19 pandemic**

Stuart Ross^1^, George Breckenridge^1^, Mengdie Zhuang^2^, Ed Manley^1,3,*^

Corresponding Author: Ed Manley e.j.manley@leeds.ac.uk

**1 England COVID-19 household visitation policies**

Table of policy regulations imposed in England during the COVID-19 pandemic, with a particular focus on household visitation policy. Information provided by the UK Commons Library^6^.

| **Period** | **Dates** | **Regulations**** |
| --- | --- | --- |
| National Lockdown 1 | 23/03/20 - 12/05/20 | Stay at Home order, to not leave home without “reasonable excuse” in law. No house visits permitted, indoors or outdoors. |
| National Lockdown 1 *easing* | 13/05/20 - 03/07/20 | **13/05/20:** Stay at Home order lifted. Recreation or unlimited exercise outdoors permitted with 1 member from another household. Encouraging workers to return to workplace where work from home not possible.  **01/06/20:** Outdoor house visits to private gardens permitted in groups of up to 6 people. Indoor house visits still not permitted unless to momentarily access outdoor private gardens. Exemptions apply. Schools begin to reopen in phased manner.  **15/06/20:** ‘Support bubble’* measures introduced to allow adults living alone (or as a single parent family household) to join, exclusively, with another ‘household’, to create a ‘linked household’ for purposes of COVID-19 gathering restrictions. Non-essential retail opens and managed return of sports events. |
| Summer 2020 | 04/07/20 – 13/10/20 | **04/07/20**: Indoor house visits permitted between 2 households*. Outdoor gatherings (e.g. private gardens) allowed up to 30 people. ‘Staycation’ accommodation in England reopens, alongside pubs and restaurants. Weddings up to 30 guests.  **14/09/20**: New ‘Rule of Six’ imposed in England, limiting social contact to 6 people in any setting, including private homes.  **24/09/20**: 10pm closing time curfew introduced for hospitality venues. |
| Leicester ‘Local Lockdown’ | 04/07/20 - 13/10/20 | No house visit gatherings permitted in Leicester City, either indoors or outdoors in private gardens.* Private house visits allowed only outdoors in private gardens upon Tier 2 entry as the Tiered System 1 began **14/10/20**. |
| Liverpool ‘Local Lockdown’ | 22/09/20 – 13/10/20 | No house visit gatherings permitted within the perimeter of Liverpool City Council, either indoors or outdoors in private gardens.* Rule of 6 in public spaces but residents advised to not socialise between households in public venues either. Liverpool City region enters toughest Tier 3 as Tiered System 1 began **14/10/20**, extending restrictions to ban (almost) all house visits. |
| Tiered System 1 | 14/10/20 – 04/11/20 | Tier 1: Indoor and outdoor house visits permitted.  Tier 2: Indoor house visits banned, outdoor house visits to private gardens permitted.*  Tier 3: No house visits permitted, indoors or outdoors.* |
| National Lockdown 2 | 05/11/20 – 01/12/20 | Stay at Home order. No indoor or outdoor house visits permitted.* |
| Tiered System 2 | 02/12/20 – 05/01/21 | Tier 1: Indoor and outdoor house visits permitted. [only Cornwall, IoS, IoW]  Tier 2: Indoor house visits banned, outdoor house visits to private gardens permitted.*  Tier 3: No house visits permitted, indoors or outdoors.*  Tier 4 [introduced 20/12/20]: Stay at Home order. No house visits permitted, indoors or outdoors.* |
| Christmas Day 2020 | 25/12/20 | ‘Christmas bubble’ permitted for 3 households to mix indoors in private homes for areas of England in Tiers 1, 2, or 3. Tier 4 areas in East and South-East England not permitted.* |
| National Lockdown 3 | 06/01/21 – 07/03/21 | Stay at Home order. No house visits permitted, indoors or outdoors.* |
| National Lockdown 3 *easing* | 08/03/21 – 19/07/21+ | **08/03/21**: Stay at Home order lifted. Schools and practical university courses return. Outdoor recreation with 1 member from another household becomes permitted. No house visits permitted, indoors or outdoors.*  **29/03/21**: Outdoor house visits in private gardens become permitted, under Rule of 6 or 2 (6 people or 2 households max.).  **17/05/21**: Indoor house visits permitted, including overnight stays, under Rule of 6 or 2. Outdoor house visit gathering limit increased to max. 30 people. |

* Unless exempt through ‘Support Bubble’ measures first introduced on 15^th^ June 2020, which allowed adults living alone to be part of another exclusive household.

** Throughout the English pandemic a range of varying exemptions were included in legislation to permit house visits. These typically included visit purposes such as caring for a vulnerable person, providing medical care, fulfilling a legal obligation, buying or moving into a new property, visiting a terminally ill relative on their deathbed, and where necessary for the delivery of essential work (e.g., fixing utilities) or education (e.g., students returning home from university).

**2 Validation against Google Mobility data**

Comparison of 7-day averaged $H_{England, t}$ and Mobility Changes from the Google COVID-19 Community Mobility Report^1^ between March 1^st^ 2020 to May 24^th^ 2021.

**
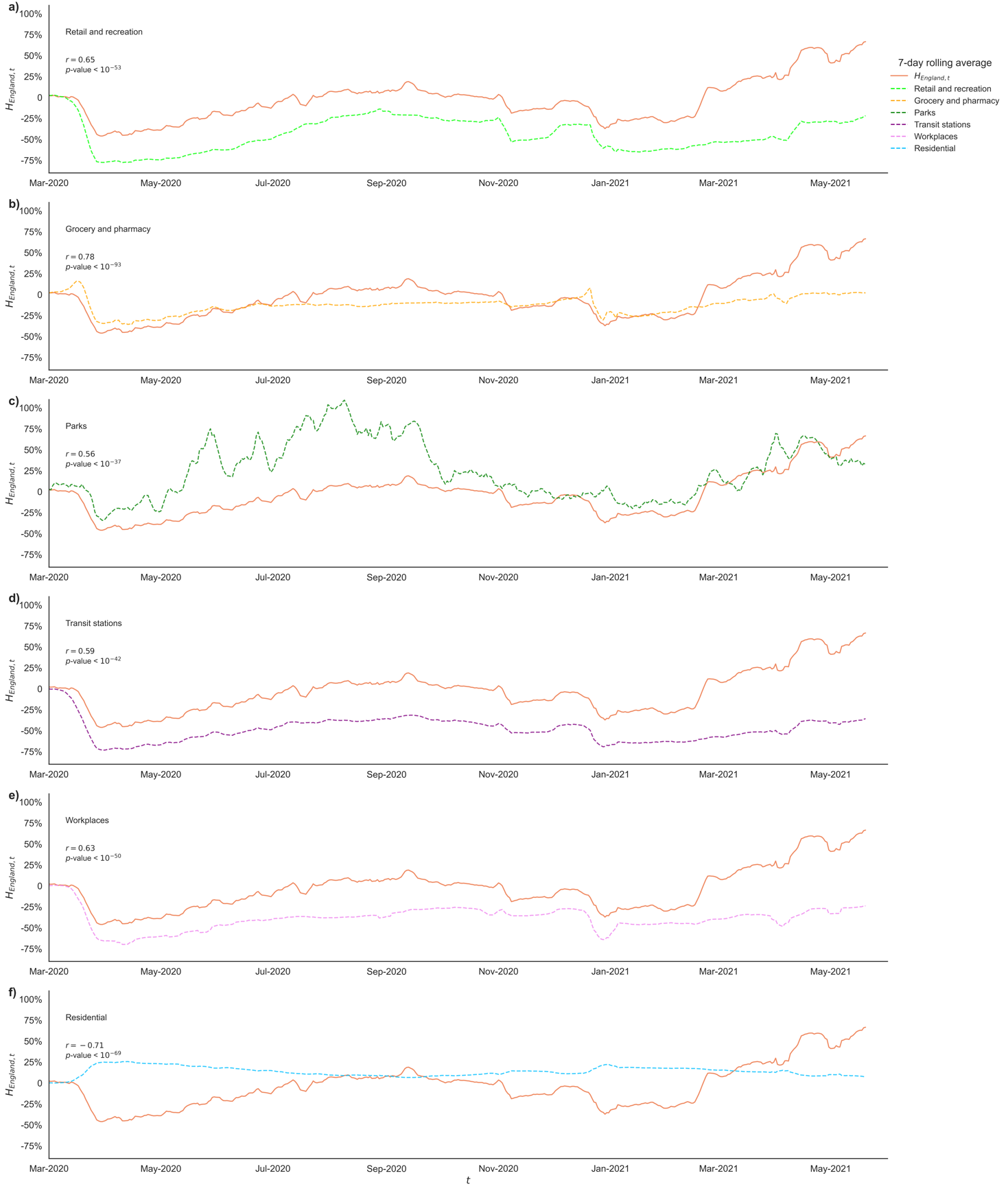
**

**3 Geographic Representativeness**

To ensure the representativeness of our data to the wider population, we validate our sample of users using a similar methodology conducted by Pepe et al. (2020) for Italy and Santana et al. (2020a) for the UK. Here, the recorded proportion of Cuebiq users in our dataset overall per area is compared to the equivalent proportion recorded by the ONS 2019 Mid-Year Population Estimates for England, across a range of geographical (regions, counties, LTLAs, and Medium Super Output Areas) and temporal scales (day, week, and month) of aggregation. We also record the proportion of total area polygons ‘dropped’ for not meeting the 10-user statistical disclosure control threshold per aggregation.

We do this analysis firstly across all Cuebiq data for England, calculating a given user’s ‘area of belonging’ by taking the centroid of their daily centroids over the given time period in January-February 2020 (see Figure S3A). These results evidence the potential granularity and excellent overall representativeness of Cuebiq data across multiple scales, demonstrating that without filtering unique users, LTLA spatial scale is the smallest unit of common spatial aggregation for which a significant ($p$=0.01, ‘**’) and highly positive correlation coefficient can be calculated across the three time periods evaluated (daily: 0.863**; weekly: 0.820**; monthly: 0.786**).

We then subsequently examine correlations in these proportions using 4 weekly sets of our total ‘active user’ sample: the set of active users used in the main study, for which we also have an associated home and work location assigned. At LTLA scale, the correlation coefficient between our user sample and ONS population proportions exceeds r = 0.86 and is significant at the 99% confidence interval across all 4 weeks. The results therefore show an excellent positive correlation between our Cuebiq data samples and the real-world proportions of residents, indicating that our Cuebiq sample is well stratified overall across English geographies and does not require artificial weighting to correct. Nevertheless, the maps below also indicate a consistent and distinct geography to Cuebiq overrepresentation, across all aggregations, concentrated in South-East England.

In Fig. S3B, the January 2020 user sample was significantly correlated with the May 2021 user sample with r=0.926**, indicating a spatial consistency in the manner in which our user base was geographically well-stratified across England throughout the period.


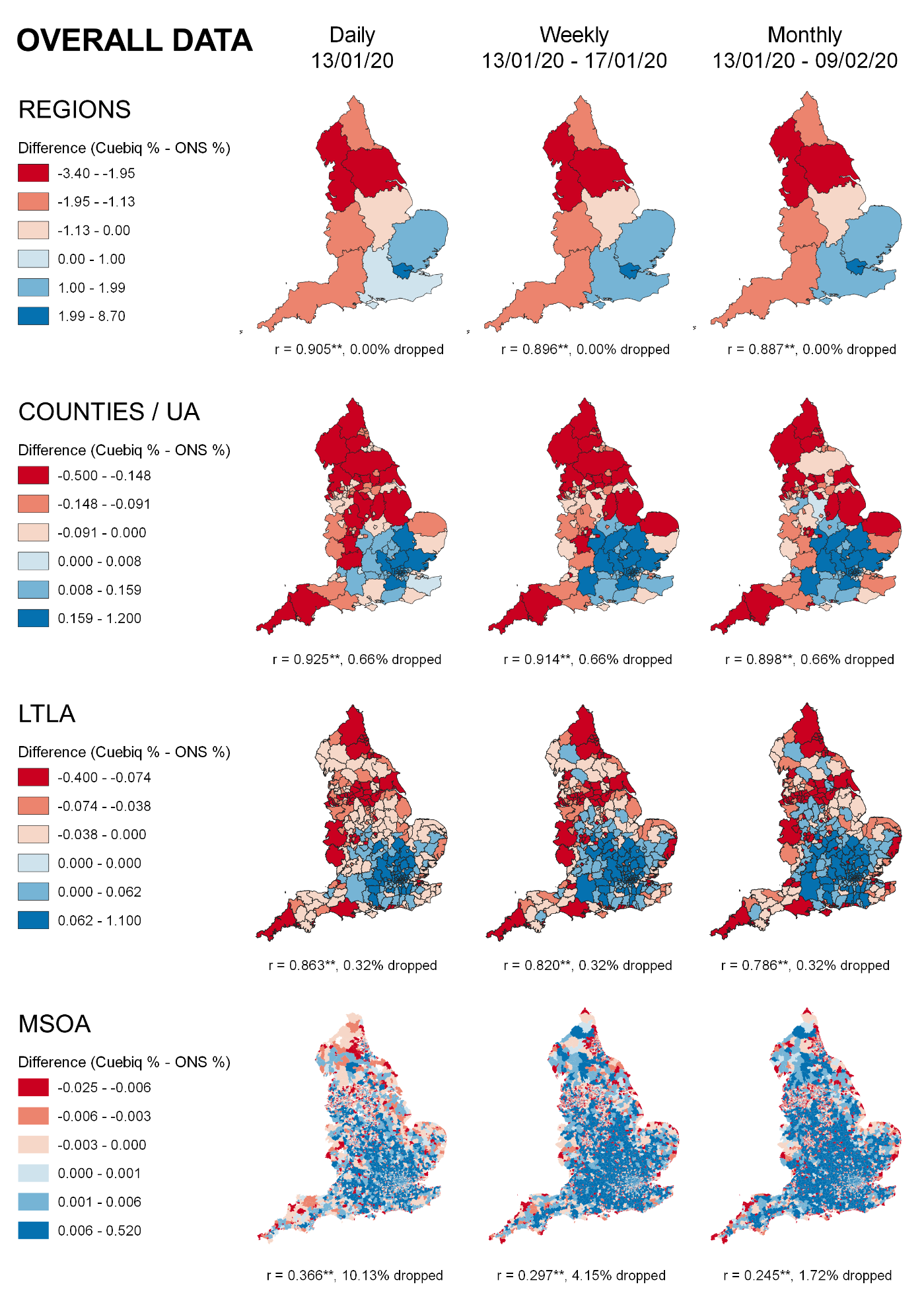


**Figure S3A**


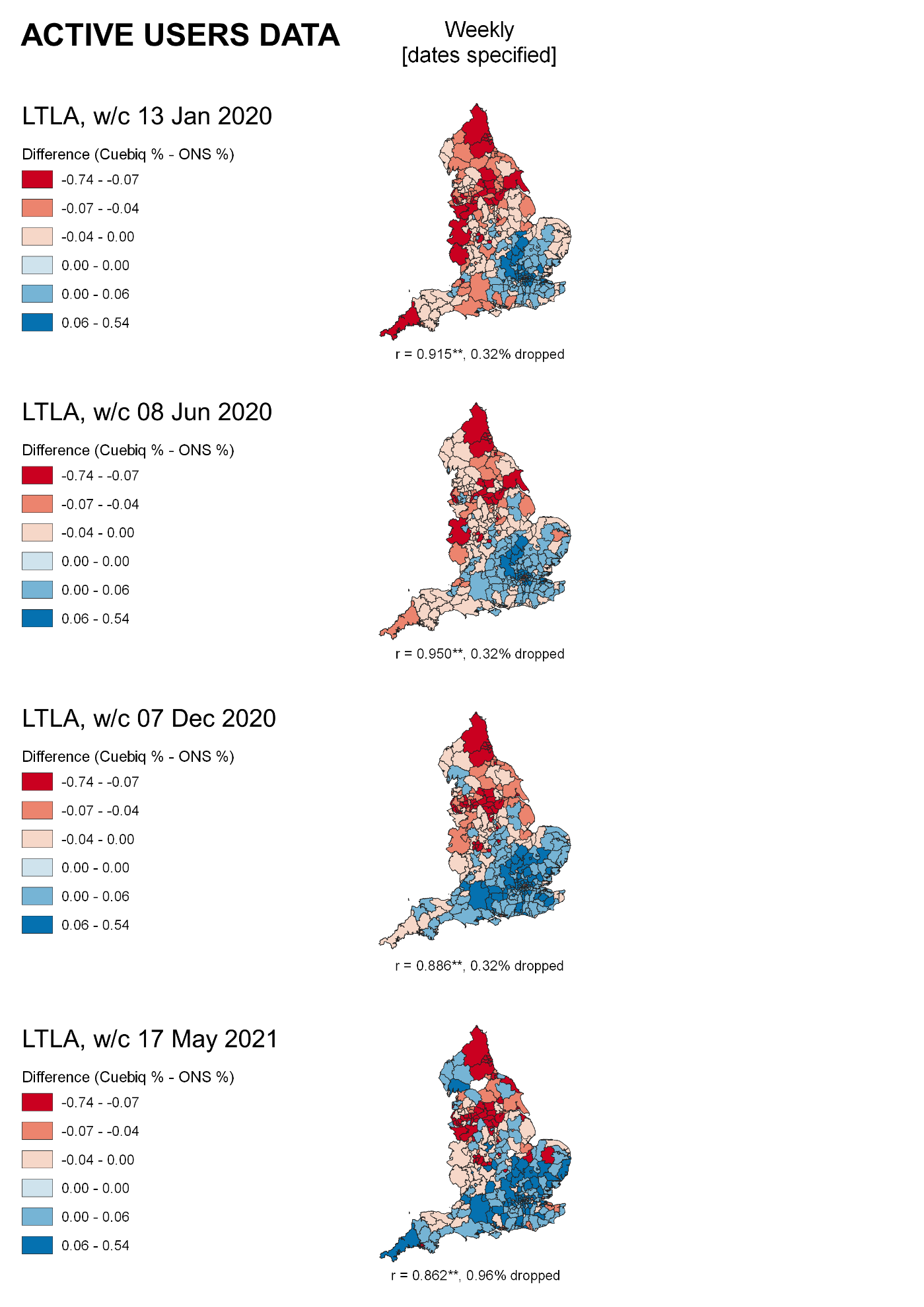

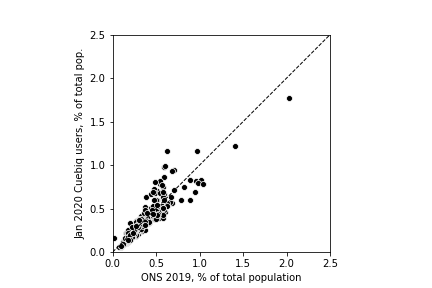

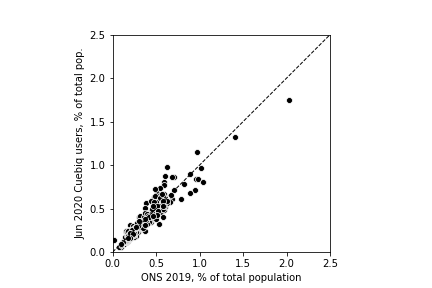

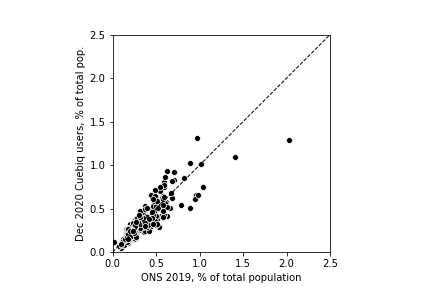

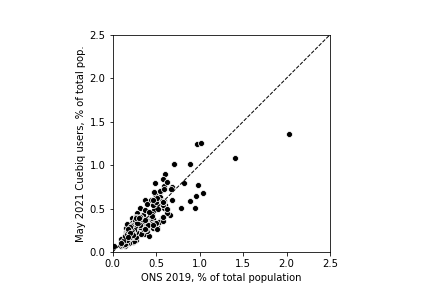


**Figure S3B**

**4 Active User Counts over Study Period**

Figure showing the numbers of active users during the study period.


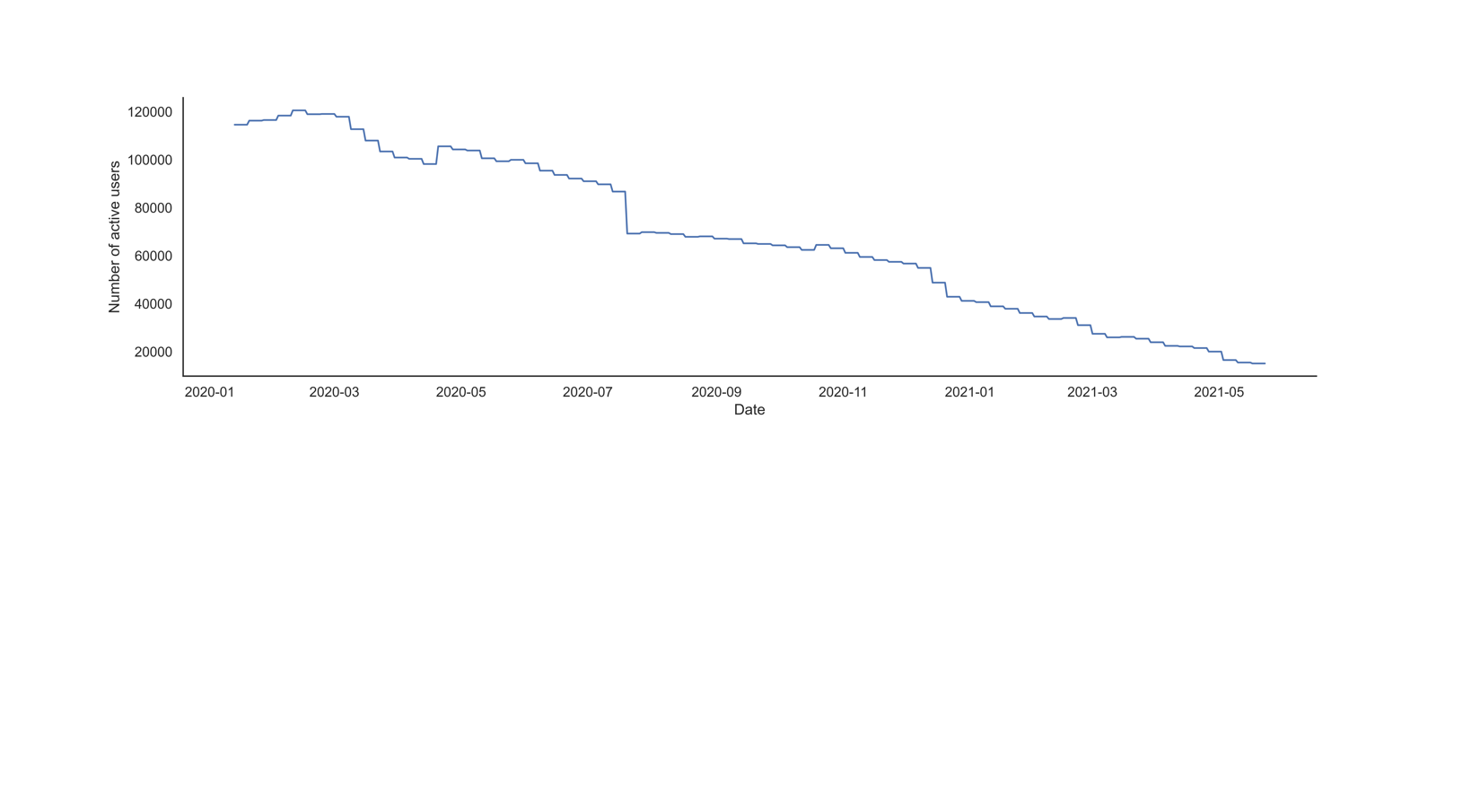

Supplement: Supplementary file 1 — Supplementary Information. [file 41598_2021_2092_MOESM1_ESM.docx]
